# Etiological shifts and clinical outcomes of acute pancreatitis between urban and rural areas: evidence from a 20-year retrospective database

**DOI:** 10.3389/fmed.2025.1640267

**Published:** 2025-07-17

**Authors:** Ximei Cao, Zide Liu, Jingwen Rao, Jie Wu, Xin Huang, Liang Xia, Lingyu Luo, Xu Shu, Yin Zhu, Nonghua Lu, Wenhua He

**Affiliations:** ^1^Department of Gastroenterology, The First Affiliated Hospital, Jiangxi Medical College, Nanchang University, Nanchang, China; ^2^Department of Gastroenterology, Jiujiang City Key Laboratory of Cell Therapy, Jiujiang No. 1 People’s Hospital, Jiujiang, China

**Keywords:** acute pancreatitis, urban–rural disparities, biliary, hypertriglyceridemic, severity propensity

## Abstract

**Background:**

Acute pancreatitis (AP) is a well-recognized digestive emergency with established clinical significance. However, current evidence regarding urban–rural distribution patterns of AP patients remains relatively limited. Through large-scale data analysis, this study aims to provide preliminary epidemiological references for this understudied area.

**Methods:**

This 20-year retrospective cohort study (2005–2024) analyzed 12,214 acute pancreatitis (AP) cases from a tertiary medical center to investigate urban–rural disparities in etiology and clinical outcomes. Patients were stratified into urban (*n* = 5,002) and rural (*n* = 7,212) groups based on residential location. We compared demographic characteristics, etiological distributions, disease severity, complications, and hospitalization outcomes between the groups. Risk factors for moderate-to-severe AP were assessed using multivariable logistic regression, with adjustment for demographic, clinical, and temporal covariates.

**Results:**

Urban patients exhibited a rising burden of hypertriglyceridemia-induced AP (HTG-AP; 30.6% vs. rural 26.3%, *p* < 0.001), surpassing biliary AP as the dominant etiology by 2023, while rural populations maintained higher biliary AP prevalence (56.4% vs. 51.7%, *p* < 0.001). Rural patients demonstrated prolonged symptom-to-admission intervals (median 3 vs. 2 days), elevated APACHE II scores (8 vs. 7), and increased severe AP incidence (20.7% vs. 18.3%, *p* < 0.01), with higher risks of infected pancreatic necrosis (5.3% vs. 4.3%) and abdominal compartment syndrome (1.7% vs. 1.1%). Multivariable analysis suggested that rural group may be associated with increased risk of moderate-to-severe AP (aOR = 1.13, *p* = 0.005), alongside hypertriglyceridemia (aOR = 2.06) and delayed admission (aOR = 1.01/day). Temporal trends revealed accelerated HTG-AP growth post-2020 in both groups, paralleling metabolic syndrome escalation.

**Conclusion:**

These findings underscore the imperative for dual interventions: urban-focused metabolic risk mitigation and rural-targeted biliary disease management, informed by evolving etiological landscapes.

## 1 Introduction

Acute pancreatitis (AP) is one of the most common critical illnesses in the digestive system, with a global annual incidence of approximately 34 per 100,000 population (1), showing an increasing trend year by year. AP has a complex etiology with highly variable clinical outcomes. Approximately 20% of patients may progress to severe acute pancreatitis (SAP), a condition associated with multiple organ failure and mortality rates reaching 30% (2, 3). In recent years, with changes in dietary patterns and the rising prevalence of metabolic diseases, the etiological spectrum of AP has undergone significant shifts. In China, hypertriglyceridemia (HTG) has nearly surpassed or even exceeded alcohol consumption as the second most common cause of AP, following biliary etiology (4–6). However, the epidemiological characteristics and clinical outcomes of AP exhibit notable regional heterogeneity, particularly between urban and rural areas with significant disparities in healthcare resource allocation and lifestyle (7). Previous studies have suggested that the etiology, severity, and clinical outcomes of AP may be influenced by multiple factors, including demographic characteristics, underlying diseases, laboratory findings, healthcare accessibility, and health behaviors (1, 2, 8–10).

Against the backdrop of rapid urbanization in China, systematic differences exist between urban and rural residents in dietary patterns (e.g., carbohydrate-dominant diets in rural areas vs. high-fat diets in urban areas), health behaviors (e.g., alcohol consumption, physical activity levels), and healthcare accessibility (e.g., primary care capacity, referral efficiency) (11, 12). The role of urban–rural disparities in the epidemiological features and clinical outcomes of AP has attracted considerable attention. Significant differences in dietary habits, lifestyle, and healthcare resource distribution between urban and rural populations may influence the occurrence and prognosis of AP (12–14). For instance, high-fat diets and alcohol intake are major risk factors for AP, and these factors are more prevalent among urban residents (15, 16). However, rural patients may face challenges such as insufficient medical resources and delayed treatment, which could adversely affect disease management and therapeutic outcomes (17).

This study, based on a prospectively maintained large-sample AP database, presents the first longitudinal comparison over 20 years of demographic characteristics, etiological composition, and clinical outcomes between urban and rural hospitalized AP patients, aiming to provide evidence for optimizing region-specific diagnostic and therapeutic strategies.

## 2 Materials and methods

### 2.1 Data source

This study was a retrospective single-center cohort study utilizing data from a prospectively maintained AP inpatient database at the First Affiliated Hospital of Nanchang University. The database was established by trained clinicians through structured electronic medical record (EMR) extraction and independently verified by two researchers to ensure data accuracy and completeness. It encompasses comprehensive information, including demographic characteristics, laboratory findings, radiological reports, AP scoring systems (e.g., APACHE II, BISAP), clinical features, and discharge outcomes. All methods were performed in accordance with the relevant guidelines and regulations, and in compliance with the Declaration of Helsinki. The study was approved by the Medical Ethics Research Committee of the First Affiliated Hospital of Nanchang University.

### 2.2 Study population

**Figure 1:**
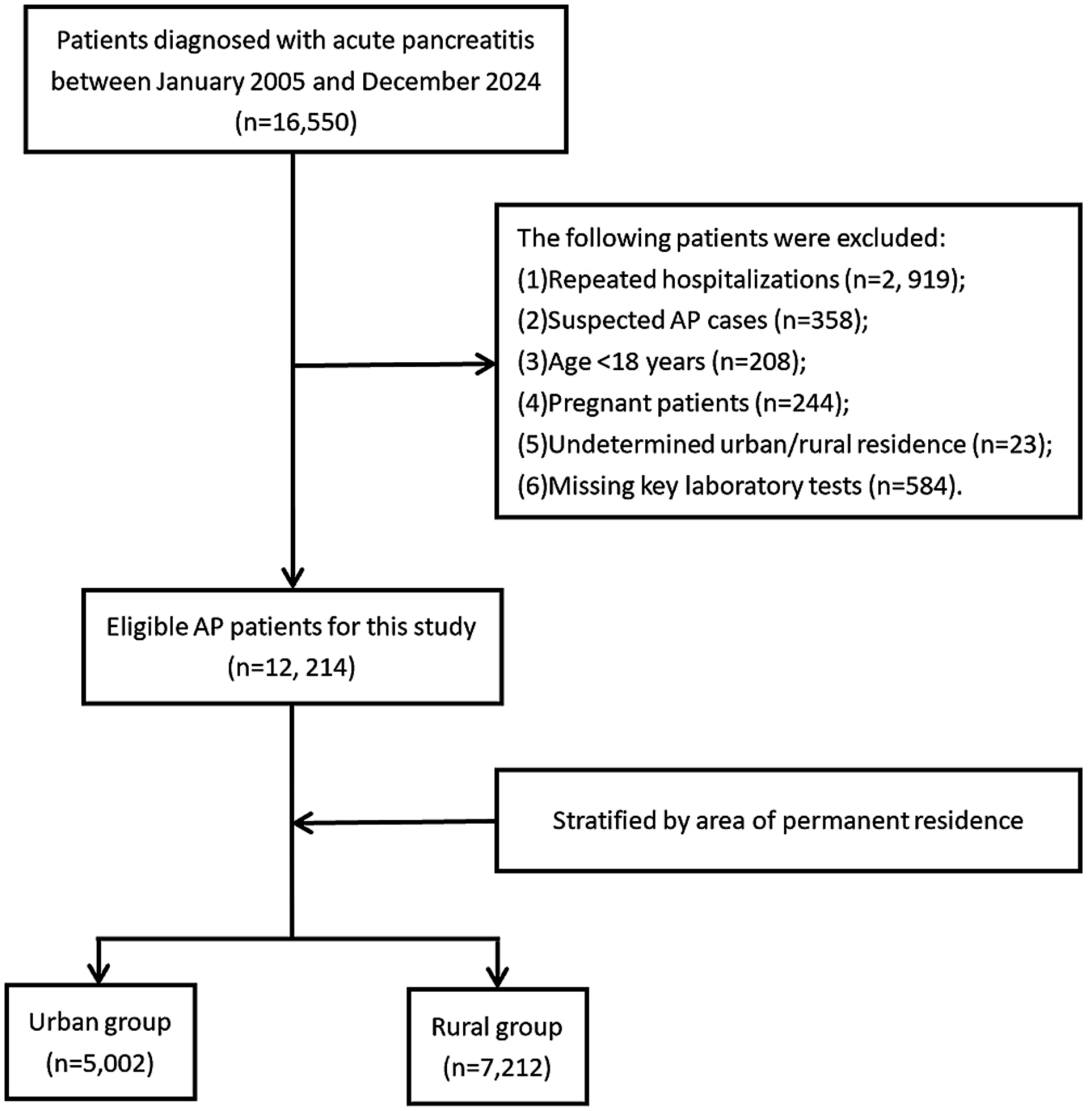
Flow chart of the patient selection process.

A total of 16,550 patients discharged with a diagnosis of acute pancreatitis (AP) from the First Affiliated Hospital of Nanchang University between January 2005 and December 2024 were initially identified. Hospital admission data for patients with AP were retrieved using the International Classification of Diseases, 10th Revision (ICD-10) codes K85-K85.92. AP diagnosis and severity classification followed the 2012 revised Atlanta criteria (18). Exclusion criteria included: (1): non-index admission for AP (prior AP hospitalization), (2), suspected AP cases, (3) age <18 years, (4) pregnancy, (5) undetermined urban–rural residency, and (6) missing critical laboratory parameters. After screening, 12,214 eligible AP patients were enrolled and stratified into urban (*n* = 5,002) and rural (*n* = 7,212) groups based on residential status. Figure 1 depicts the participant selection procedure.

### 2.3 Urban–rural classification

Patients were stratified into urban or rural groups based on residential duration and geographical criteria: urban residency was defined as permanent residence (≥6 months/year) in prefecture-level cities or municipal districts, while rural residency encompassed townships or administrative villages. Residential data were extracted from hospitalization records, which mandated patients to report detailed addresses and contact information. For migrant populations, classification prioritized actual residential address over household registration (e.g., rural-registered individuals residing long-term in urban areas were categorized as urban). Additionally, health insurance type (urban employee/resident insurance vs. new rural cooperative medical scheme) and occupation (agricultural vs. non-agricultural) were used as secondary reference criteria. Cases with indeterminate residency (e.g., temporary migrant workers lacking fixed addresses) were excluded to minimize misclassification bias. This approach ensured alignment between residential exposure and epidemiological risk profiles.

### 2.4 Data extraction

Data collection encompassed five domains: (1) Demographics—age, gender, residential status, smoking, and alcohol consumption; (2) Baseline disease characteristics—subclassified into etiology (biliary, HTG, alcoholic, idiopathic, drug-induced, post-surgical, traumatic, post-ERCP, autoimmune, parapapillary diverticulum of duodenum, or other), severity (mild, moderately severe, or severe AP per revised Atlanta criteria), comorbidities (hypertension, diabetes mellitus, hyperlipidemia, chronic obstructive pulmonary disease [COPD], coronary heart disease, cirrhosis, cerebral infarction, chronic renal failure), and admission clinical status (fever on admission, APACHE II score within 24 h); (3) Treatment process—including healthcare access (prior hospital transfer) and time metrics (symptom-to-admission interval); (4) Laboratory parameters—white blood cell count (×10^9^/L) and serum amylase (U/L); (5) Clinical outcomes—stratified into short-term outcomes (in-hospital mortality, total hospital stay, ICU stay, hospitalization costs) and complications (local: infected pancreatic necrosis, abdominal compartment syndrome, enteric fistula; systemic: acute respiratory failure, acute kidney injury, circulatory failure, septic shock, heart failure, hepatic dysfunction; thrombotic: portosplenomesenteric venous thrombosis, deep vein thrombosis). This classification aligns with acute pancreatitis research frameworks, ensuring logical separation of intrinsic disease attributes from care-related factors.

### 2.5 Statistical analysis

All statistical analyses were performed using R software (version 4.4.1). Missing data were handled using multiple imputation with chained equations (MICE), generating 20 imputed datasets based on all analysis variables. Continuous variables were evaluated for normality using Shapiro–Wilk tests and presented as mean ± standard deviation (SD) for normally distributed data or median (interquartile range, IQR) for non-normally distributed data. Categorical variables were expressed as frequencies (percentages). Group comparisons were conducted using independent t-tests (for normally distributed continuous variables), Mann–Whitney U tests (for non-normally distributed continuous variables), or chi-square/Fisher’s exact tests (for categorical variables), as appropriate.

Continuous variables that violated linearity assumptions were categorized into clinically meaningful groups: hospitalization costs (≤11,250, 11,251-26,270, >26,270 Yuan), APACHE II scores (<8, 8–12, ≥12), and white blood cell counts (≤10, 10.1–20, >20 × 10^9^/L). Multivariable logistic regression models were developed to identify risk factors, incorporating variables with *p* < 0.1 in univariate analysis or clinical significance (Supplementary Table 1). Multicollinearity was assessed using variance inflation factors (VIF < 5 for all retained variables). Model performance was evaluated by calculating area under the receiver operating characteristic curve (AUC). Temporal trends were analyzed using 5-year intervals (2005–2009 to 2020–2024). Results were presented as adjusted odds ratios with 95% confidence intervals and visualized in forest plots. All tests were two-tailed with *p* < 0.05 considered statistically significant.

## 3 Results

### 3.1 Demographic and clinical characteristics

This study enrolled 12,214 acute pancreatitis patients, comprising 5,002 urban cases (40.9%) and 7,212 rural cases (59.1%). Comparative analysis revealed urban group had significantly higher proportions of males, younger age, greater alcohol, and higher prevalence of hypertension, diabetes, and hyperlipidemia (all *p* < 0.05). Rural patients demonstrated significantly higher rates of prior hospital transfer, longer symptom-to-admission intervals, elevated APACHE II scores, and greater severe acute pancreatitis incidence (all *p* < 0.01). While rural patients incurred higher hospitalization costs, no significant differences existed in length of stay or mortality. Temporal trends showed rural case proportions increased markedly post-2015 (rising from 8.6% in 2005–2009 to 36.5% in 2015–2019), whereas urban cases peaked during 2020–2024. Baseline clinical characteristics of the two groups are detailed in Table 1.

**Table 1:** Baseline characteristics of patients with acute pancreatitis between urban and rural groups.

Variable	Total (*n* = 12,214)	Urban (*n* = 5,002)	Rural (*n* = 7,212)	*p*-value
Gender, *n* (%)				<0.001
Male	7,284 (59.6)	3,182 (63.6)	4,102 (56.9)	
Female	4,930 (40.4)	1,820 (36.4)	3,110 (43.1)	
Age, [IQR], (y)	51.00 [40.00, 64.00]	50.00 [39.00, 63.00]	52.00 [40.00, 64.00]	0.002
Smoking, *n* (%)	3,221 (26.4)	1,351 (27.0)	1,870 (25.9)	0.19
Alcohol consumption, n (%)	3,315 (27.1)	1,423 (28.4)	1,892 (26.2)	0.007
Comorbidities, *n* (%)				
Hypertension	2,347 (19.2)	1,022 (20.4)	1,325 (18.4)	0.005
Diabetes mellitus	1,282 (10.5)	568 (11.4)	714 (9.9)	0.011
Hyperlipidemia	892 (7.3)	403 (8.1)	489 (6.8)	0.009
COPD	114 (0.9)	47 (0.9)	67 (0.9)	1
Coronary heart disease	127 (1.0)	58 (1.2)	69 (1.0)	0.319
Cirrhosis	39 (0.3)	18 (0.4)	21 (0.3)	0.618
Cerebral infarction	184 (1.5)	78 (1.6)	106 (1.5)	0.746
Chronic renal failure	47 (0.4)	14 (0.3)	33 (0.5)	0.158
Clinical characteristics				
Prior transfer, *n* (%)				<0.001
Yes	8,643 (70.8)	3,202 (64.0)	5,441 (75.4)	
No	3,571 (29.2)	1,800 (36.0)	1,771 (24.6)	
Fever on admission, [IQR] (°C)	36.85 [0.85]	36.90 [0.90]	36.80 [0.80]	0.058
Days to admission, [IQR]	3.00 [1.00, 6.00]	2.00 [1.00, 5.00]	3.00 [1.00, 6.00]	<0.001
WBC [IQR] (×10⁹/L)	11.54 [8.00, 15.22]	11.59 [8.10, 15.00]	11.50 [8.00, 15.42]	0.93
AMY [IQR] (U/L)	212.00 [76.00, 684.78]	223.90 [76.08, 736.38]	203.95 [76.00, 649.00]	0.005
APACHE II score, [IQR]	8.00 [5.00, 11.00]	7.00 [4.00, 11.00]	8.00 [5.00, 11.00]	<0.001
Disease severity, *n* (%)				<0.001
MAP	5,536 (45.3)	2,421 (48.4)	3,115 (43.2)	
MSAP	4,268 (34.9)	1,665 (33.3)	2,603 (36.1)	
SAP	2,410 (19.7)	916 (18.3)	1,494 (20.7)	
Hospital stays, [IQR] (days)	8.00 [6.00, 14.00]	8.00 [6.00, 13.00]	9.00 [6.00, 14.00]	0.059
ICU stay, mean ± SD (days)	2.56 ± 0.070	2.44 ± 0.108	2.64 ± 0.093	0.012
Hospitalization costs, [IQR] (CNY)	17,199.67 [9,232.59, 34,380.93]	15,753.47 [8,585.58, 32,420.09]	18,369.00 [9,668.40, 35,632.40]	<0.001
Mortality, *n* (%)	235 (1.9)	84 (1.7)	151 (2.1)	0.116
Time period, *n* (%)				<0.001
2005–2009	1,299 (10.6)	676 (13.5)	623 (8.6)	
2010–2014	2,182 (17.9)	904 (18.1)	1,278 (17.1)	
2015–2019	3,967 (32.5)	1,332 (26.6)	2,635 (36.5)	
2020–2024	4,766 (39)	2090 (41.8)	2,676 (37.1)	

AMY, amylase; APACHE II, Acute Physiology and Chronic Health Evaluation II; COPD, Chronic obstructive pulmonary disease; IQR, interquartile range; MAP, mild acute pancreatitis; MSAP, moderately severe acute pancreatitis; SAP, severe acute pancreatitis; WBC, White Blood Cell; ICU, Intensive Care Unit; CNY, Chinese Yuan. The data collection time points for WBC, AMY, and APACHEII were within 24 h of admission.

### 3.2 Systemic complications

As shown in Table 2, acute respiratory failure and acute kidney injury were the most common systemic complications overall, with no significant urban–rural differences (both *p* > 0.05). Notably, rural patients exhibited significantly higher rates of circulatory failure (3.7% vs. 2.9%, *p* = 0.022), abdominal compartment syndrome (1.7% vs. 1.1%, *p* = 0.011), and infected pancreatic necrosis (5.3% vs. 4.3%, *p* = 0.014). In contrast, other complications including septic shock, heart failure, and thrombotic events showed no statistically significant intergroup differences.

**Table 2:** Composition of complications between urban and rural groups with acute pancreatitis.

Complication	Total (*n* = 12, 214)	Urban (*n* = 5, 002)	Rural (*n* = 7, 212)	*p*-value
Acute respiratory failure, *n* (%)	2, 014 (16.5)	794 (15.9)	1, 220 (16.9)	0.133
Acute kidney failure, *n* (%)	640 (5.2)	255 (5.1)	385 (5.3)	0.586
Circulatory failure, *n* (%)	410 (3.4)	145 (2.9)	265 (3.7)	0.022
Septic shock, *n* (%)	175 (1.4)	61 (1.2)	114 (1.6)	0.115
Heart failure, *n* (%)	40 (0.3)	21 (0.4)	19 (0.3)	0.185
Liver dysfunction, *n* (%)	769 (6.3)	305 (6.1)	464 (6.4)	0.475
Abdominal compartment syndrome, *n* (%)	181 (1.5)	57 (1.1)	124 (1.7)	0.011
Portal/splenic/mesenteric vein thrombosis, *n* (%)	134 (1.1)	52 (1.0)	82 (1.1)	0.675
Deep vein thrombosis of the lower limbs, *n* (%)	39 (0.3)	16 (0.3)	23 (0.3)	1
Intestinal fistula, *n* (%)	87 (0.7)	33 (0.7)	54 (0.7)	0.641
Infected necrotizing pancreatitis, *n* (%)	594 (4.9)	214 (4.3)	380 (5.3)	0.014

### 3.3 Etiology of AP

As shown in Table 3, biliary, HTG, and alcoholic etiologies constituted the top three causes of AP in both urban and rural populations. Biliary AP was the most prevalent (54.5%), with significantly higher incidence in rural patients (56.4% vs. 51.7%, *p* < 0.001). HTG-AP accounted for 28.1% of cases, demonstrating a higher proportion in urban areas (30.6% vs. 26.3%, *p* < 0.001). No significant urban–rural difference was observed in alcoholic AP, while idiopathic AP was more common in urban patients (8.2% vs. 6.9%, *p* = 0.010). Notably, statistically significant disparities were identified in drug-induced AP and post-ERCP AP between the two groups. Other etiologies (e.g., traumatic, autoimmune, and periampullary diverticulum-associated AP showed no significant intergroup differences).

**Table 3:** Etiological composition of acute pancreatitis between urban and rural groups.

Etiology	Total (*n* = 12, 214)	Urban (*n* = 5, 002)	Rural (*n* = 7, 212)	*p*-value
Biliary, *n* (%)	6,654 (54.5)	2, 588 (51.7)	4, 066 (56.4)	<0.001
HTG, *n* (%)	3,430 (28.1)	1, 533 (30.6)	1, 897 (26.3)	<0.001
Alcoholic, *n* (%)	952 (7.8)	398 (8.0)	554 (7.7)	0.601
Idiopathic, *n* (%)	909 (7.4)	409 (8.2)	500 (6.9)	0.01
Drug-induced, *n* (%)	30 (0.2)	5 (0.1)	25 (0.3)	0.012
Post-surgical^#^, *n* (%)	29 (0.2)	8 (0.2)	21 (0.3)	0.202
Traumatic, *n* (%)	43 (0.4)	17 (0.3)	26 (0.4)	0.973
Post-ERCP, *n* (%)	62 (0.5)	34 (0.7)	28 (0.4)	0.036
Autoimmunity, *n* (%)	36 (0.3)	14 (0.3)	22 (0.3)	0.934
Parapapillary diverticulum of duodenum, *n* (%)	40 (0.3)	19 (0.4)	21 (0.3)	0.495
Other causes*, *n* (%)	509 (4.2)	203 (4.1)	306 (4.2)	0.616

HTG, Hypertriglyceridaemia. ERCP: Endoscopic Retrograde Cholangiopancreatography. *It refers to the acute pancreatitis after surgery. †Other etiologies: pancreatic cancer, ampullary cancer, papillary sphincter dysfunction, pancreatic Division.

### 3.4 Temporal trends in the three major etiologies of AP (2005–2024)

**Figure 2:**
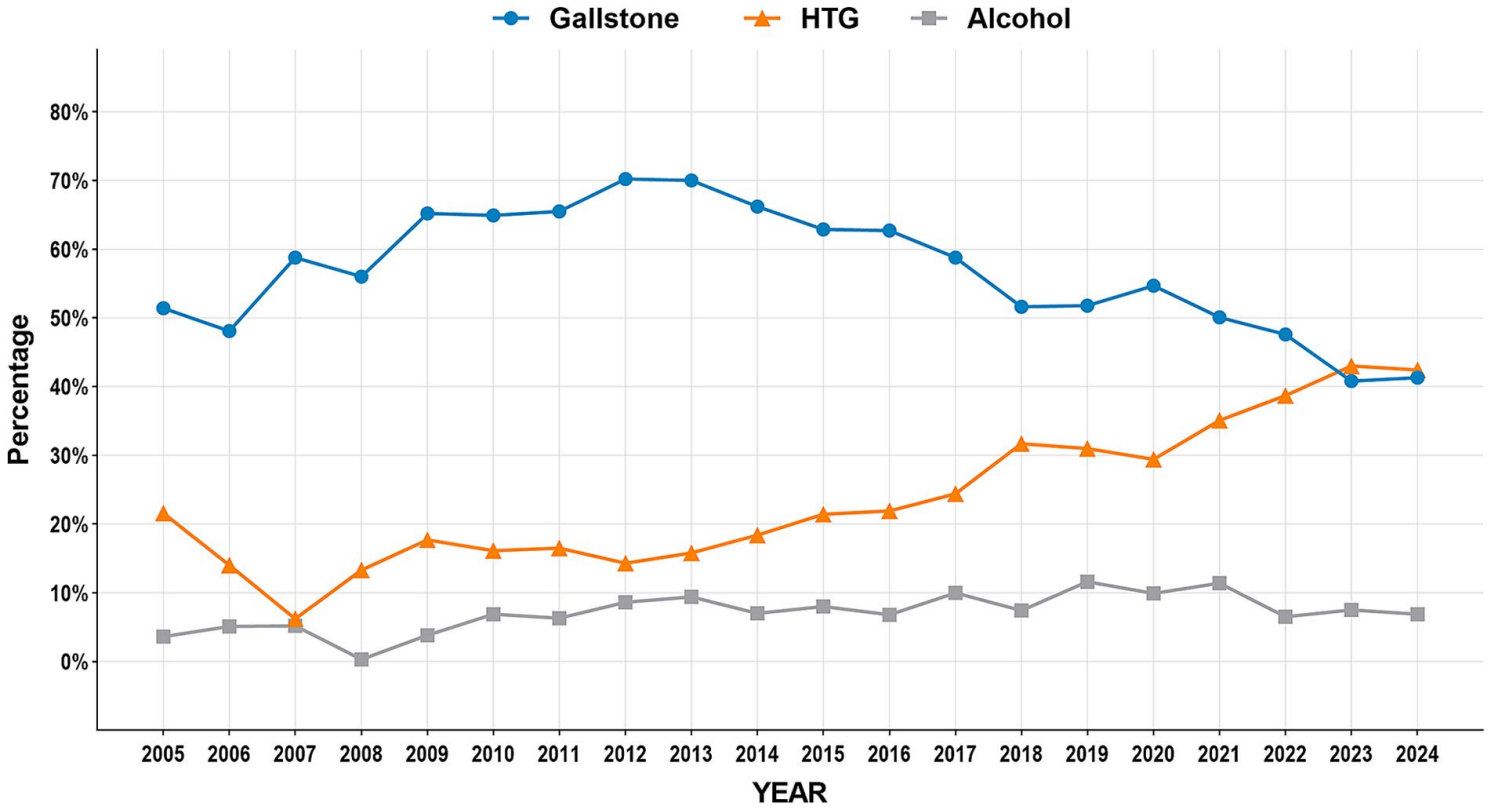
Annual variation in major etiologies of acute pancreatitis (2005–2024).

**Figure 3:**
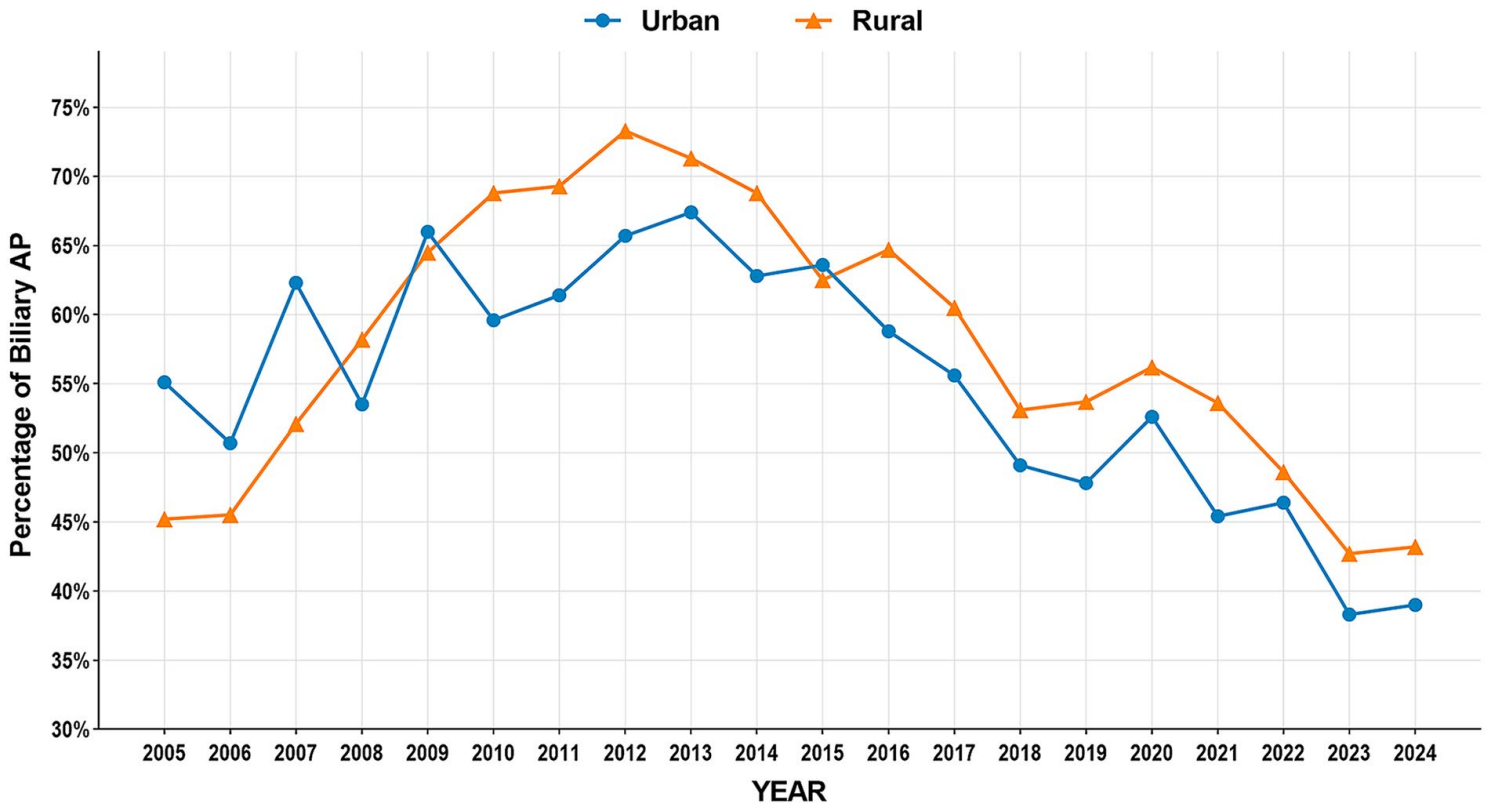
Annual proportion changes of biliary acute pancreatitis cases: Urban vs. Rural Groups.

**Figure 4:**
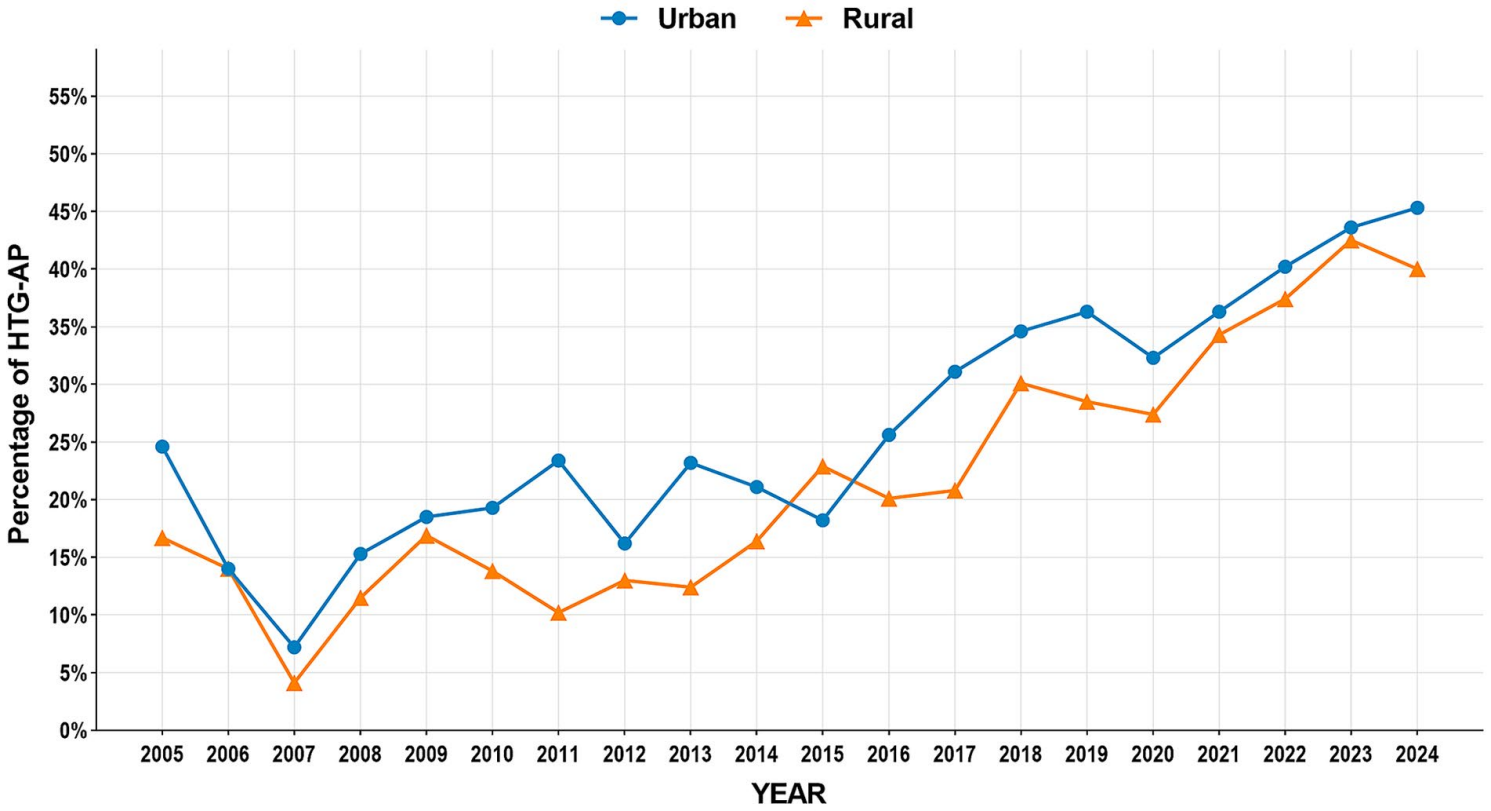
Annual proportion changes of hypertriglyceridemic acute pancreatitis cases: Urban vs. Rural Groups.

**Figure 5:**
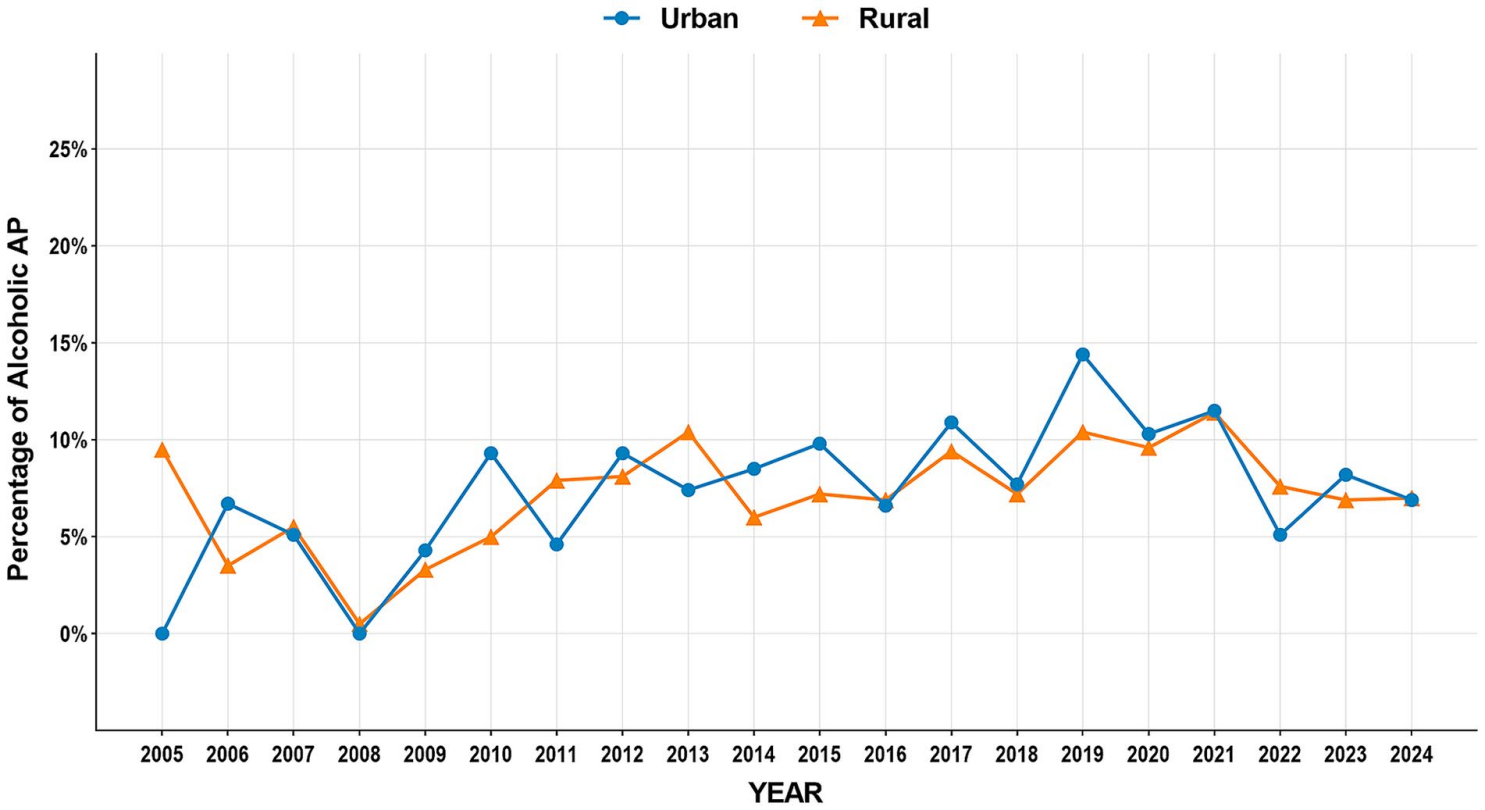
Annual proportion changes of alcoholic acute pancreatitis cases: urban vs. rural groups.

The study period witnessed significant epidemiological shifts in AP etiology (Figure 2). Biliary AP demonstrated a consistent downward trend (51.4% → 41.3%), with rural areas maintaining higher prevalence than urban areas (2024, 43.2% vs. 39.0%) (Figure 3). In contrast, HTG-AP showed remarkable growth (21.6% → 42.4%), surpassing biliary AP as the leading etiology in 2023 (43.0%). The urban group consistently demonstrated higher proportions than the rural group in most years, with both groups exhibiting a steep surge in growth rates since 2020 (Figure 4). Alcoholic AP displayed fluctuating proportions (3.6–11.6%) with diminishing urban–rural disparities (Figure 5). Importantly, the urban–rural gaps for HTG-AP and biliary AP narrowed substantially from 7.9 to 5.3% and 9.9 to 4.2%, respectively, suggesting converging epidemiological patterns.

### 3.5 Multivariable logistic regression analysis of factors associated with moderate-to-severe AP

Multivariable logistic regression analysis identified significant risk factors for moderate-to-severe AP (Table 4). As shown in the fully adjusted model (Model 3), rural group was significantly associated with increased disease severity (adjusted OR = 1.13, 95% CI: 1.04–1.24, *p* = 0.005) compared with urban residence. Hypertriglyceridemia-induced AP (HTG-AP) demonstrated the strongest association (aOR = 2.06, 95% CI: 1.79–2.38, *p* < 0.001), followed by alcoholic etiology. Clinical predictors including prior hospital transfer, delayed admission, elevated white blood cell count, and higher APACHE II scores were all significantly associated with increased disease severity (all *p* < 0.001). Notably, patients with APACHE II scores ≥12 had 2.76-fold higher risk of severe outcomes (*p* < 0.001). Higher hospitalization costs were strongly correlated with disease severity (aOR = 12.6, *p* < 0.001), likely reflecting the more intensive treatment required for severe cases. Compared with the 2005–2009 baseline period, subsequent years showed elevated risks of severe AP.

**Table 4:** Multivariable logistic regression analysis of factors associated with moderate-to-severe acute pancreatitis.

Variable	Model 1	*p*-value	Model 2	*p*-value	Model 3	*p*-value	Reference
	OR (95% CI)		aOR (95% CI)		aOR (95% CI)		
Rural group	1.23 (1.15–1.33)	<0.001	1.24 (1.16–1.34)	<0.001	1.13 (1.04–1.24)	0.005	Urban group
Female gender	–	–	1.00 (0.92–1.09)	0.95	1.01 (0.93–1.00)	0.91	Male
Age (per 10 years)	–	–	1.01 (0.99–1.04)	0.26	0.97 (0.93–1.00)	0.049	–
Smoking	–	–	1.02 (0.92–1.12)	0.76	0.96 (0.85–1.09)	0.54	Non-smoker
Alcohol consumption	–	–	1.40 (1.27–1.55)	<0.001	1.01 (0.97–1.25)	0.12	Non-drinker
Etiology							
Biliary AP	–	–	–	–	1.05 (0.933–1.169)	0.45	No
HTG-AP	–	–	–	–	2.06 (1.79–2.38)	<0.001	No
Alcoholic AP	–	–	–	–	1.93 (1.624–2.351)	<0.001	No
Clinical features							
Prior transfer	–	–	–	–	1.53 (1.39–1.69)	<0.001	Non-transfer
Days to admission	–	–	–	–	1.01 (1.01–1.02)	<0.001	–
WBC count							
Mild elevation	–	–	–	–	1.78 (1.62–1.95)	<0.001	Normal
Marked elevation	–	–	–	–	2.9 (2.41–3.51)	<0.001	Normal
APACHE II score							
8–12	–	–	–	–	1.47 (1.32–1.63)	<0.001	<8
≥12	–	–	–	–	2.76 (2.41–3.16)	<0.001	<8
Hospitalization costs							
Medium	–	–	–	–	3.20 (2.89–3.54)	<0.001	low
High	–	–	–	–	12.6(11.2–14.2)	<0.001	low
Time period							
2010–2014	–	–	–	–	1.49 (1.26–1.76)	<0.001	2005–2009
2015–2019	–	–	–	–	1.23 (1.05–1.44)	0.011	2005–2009
2020–2024	–	–	–	–	1.24 (1.06–1.46)	0.008	2005–2009

Model 1 (crude) examined the unadjusted association between rural residence and moderate-to-severe AP. Model 2 adjusted for basic demographic factors [age (per 10-year increment), sex, smoking status, and alcohol consumption]. Model 3 (fully adjusted) additionally incorporated clinical variables (prior hospital transfer, AP etiology, comorbidities, laboratory parameters, disease severity scores, hospitalization costs, and time period) that were significant (*p* < 0.1) in univariate analysis or clinically relevant. Crude and adjusted odds ratios (ORs) with 95% confidence intervals (CIs) were estimated using logistic regression models.

**Figure 6:**
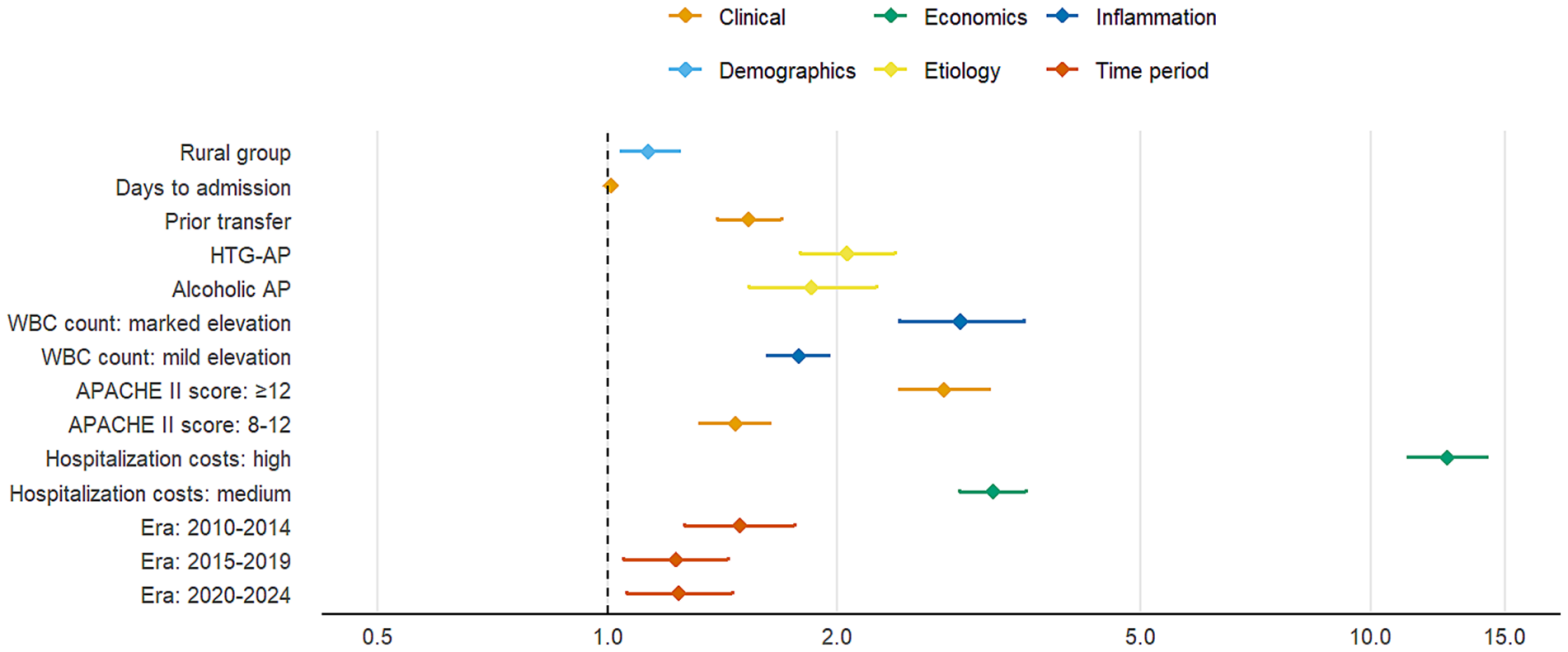
Forest plot of significant risk factors for moderate-to-severe acute pancreatitis.

**Figure 7:**
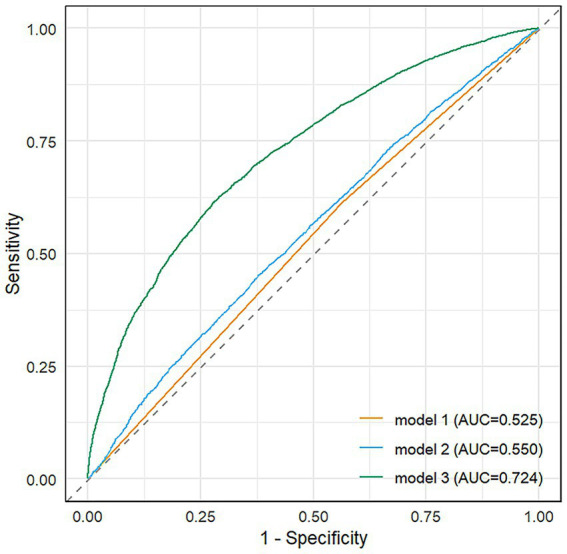
Receiver operating characteristic (ROC) curves of three prediction models.

The forest plot (Figure 6) displays adjusted odds ratios with 95% confidence intervals for all variables showing statistically significant associations with disease severity (*p* < 0.05), organized by clinical domains. The final multivariable model (Model 3) demonstrated good discriminative capacity (AUC = 0.724; Figure 7), representing a 39.8% relative improvement over the crude model (Model 1 AUC = 0.525).

## 4 Discussion

This study, based on 20-year inpatient data from a single tertiary center, reveals distinct etiological, clinical, and prognostic differences between urban and rural AP patients in the region. Despite limitations in generalizability inherent to single-center data, the large sample size and extended observational period provide valuable insights into regional urban–rural healthcare disparities.

Both urban and rural populations showed similar etiological shifts in AP, characterized by decreasing biliary cases and significantly increasing HTG-AP incidence. These findings are consistent with our prior collaborative research conducted with the North China Pancreatic Center (19). HTG-AP has emerged as the second leading etiology after gallstone disease (5, 6), with its incidence persistently increasing over the past 7 years, demonstrating notable seasonality and holiday-related peaks (20). The persistently higher prevalence of biliary AP in rural patients (56.4% vs. 51.7%) may reflect insufficient screening for biliary stones and delayed health awareness despite increasing high-fat dietary intake (21, 22). Meanwhile, the narrowing urban–rural gap in HTG-AP (from 7.9 to 5.3%) highlights emerging challenges in managing metabolic risk factors in rural areas. Additionally, the higher incidence of drug-induced AP in urban patients (0.3% vs. 0.1%) may correlate with more frequent use of medications such as antineoplastic and antibiotics (23, 24), warranting enhanced clinical vigilance in pharmacotherapy.

Despite comparable mortality rates, rural patients exhibited delayed symptom-to-admission intervals (3 vs. 2 days), higher prior hospital transfer rates (75.4% vs. 64.0%), and elevated APACHE II scores (8 vs. 7), suggesting deficiencies in early AP recognition and initial management at primary care facilities (25). Multivariable analysis revealed that rural residence may be associated with an increased risk for moderate-to-severe AP (adjusted OR = 1.13, 95% CI: 1.04–1.24), likely attributable to delays in referral and insufficient prehospital emergency resources (26, 27). Notably, rural patients had higher rates of infected pancreatic necrosis (5.3% vs. 4.3%) and abdominal compartment syndrome (1.7% vs. 1.1%), potentially linked to delayed standardized fluid resuscitation and antibiotic administration (3, 28), underscoring the need to optimize acute-phase management in hierarchical healthcare systems.

Temporal trends showed a marked rise in rural AP cases after 2015 (8.6% → 36.5%), likely driven by expanded reimbursement coverage under the New Rural Cooperative Medical Scheme and improved referral practices (29, 30). The peak severity risk during 2010–2014 (aOR = 2.23) and subsequent decline to 2020–2024 (aOR = 1.75) may reflect the dissemination of AP management guidelines and minimally invasive techniques (3, 31). However, the post-2020 surge in HTG-AP across both populations raises concerns about COVID-19-related lifestyle changes (e.g., reduced physical activity, dietary shifts) accelerating metabolic pancreatitis (32, 33), a hypothesis requiring multicenter validation.

While this study benefits from its large sample and longitudinal design, limitations persist. First, as a single-center retrospective study, selection bias may distort the true urban–rural AP distribution in the general population. Second, reliance on tertiary hospital data may overestimate disease severity, particularly for rural cases due to referral bias. Third, the lack of prehospital management details and socioeconomic factors (e.g., insurance types, income) limits mechanistic exploration. Future multicenter studies incorporating primary care data and community-based surveys are needed to clarify sociodemographic determinants of urban–rural disparities and inform targeted prevention strategies.

## 5 Conclusion

In conclusion, our study reveals that rural patients exhibit a higher prevalence of gallstone-related AP, delayed hospital visits, and more severe clinical conditions, while HTG-AP shows a rapid upward trend in both urban and rural areas, potentially linked to dietary changes. Although rural patients demonstrate higher referral rates and complication risks, recent improvements in medical care have reduced their severe AP incidence. These findings underscore the necessity to enhance primary healthcare systems and implement targeted prevention strategies.

## Data Availability

The original contributions presented in the study are included in the article/Supplementary material, further inquiries can be directed to the corresponding author/s.
