# Supplementary material for: Etiological shifts and clinical outcomes of acute pancreatitis between urban and rural areas: evidence from a 20-year retrospective database
Source: Front Med (Lausanne). 2025 Jul 17;12:1640267. doi: 10.3389/fmed.2025.1640267 (PMC12310655; doi:10.3389/fmed.2025.1640267)
Supplement: Supplementary file 1 [file Table_1.DOCX]

**Supplementary Table 1.** Multivariable logistic regression analysis of factors associated with moderate-to-severe acute pancreatitis.

| **Variable** | **OR (95% CI)** | ***P-value*** |
| --- | --- | --- |
| Rural group | 1.23 (1.15-1.33) | <0.001 |
| Female | 0.89 (0.83-0.96) | 0.002 |
| Age_per10 | 1.00 (0.98-1.02) | 0.902 |
| Smoking | 1.21 (1.11-1.31) | <0.001 |
| Alcohol consumption | 1.39 (1.29-1.51) | <0.001 |
| **Comorbidities** |  |  |
| Hypertension | 1.36 (1.24-1.49) | <0.001 |
| Diabetes mellitus | 1.35 (1.20-1.52) | <0.001 |
| Hyperlipidemia | 1.52 (1.32-1.75) | <0.001 |
| **Etiology** |  |  |
| Biliary AP | 0.73 (0.68-0.79) | <0.001 |
| HTG AP | 1.81 (1.67-1.97) | <0.001 |
| Alcoholic AP | 1.77 (1.53-2.03) | <0.001 |
| Prior transfer | 1.86 (1.72-2.02) | <0.001 |
| Days to admission | 1.01 (1.00-1.01) | <0.001 |
| AMY | 1.00 (1.00-1.00) | 0.009 |
| WBC count |  |  |
| normal | Ref (1.00) |  |
| Mild Elevation | 2.12 (1.97-2.29) | <0.001 |
| Marked Elevation | 5.36 (4.56-6.33) | <0.001 |
| APACHEII |  |  |
| ＜8 | Ref (1.00) |  |
| 8-12 | 1.70 (1.56-1.84) | <0.001 |
| ≥12 | 3.71 (3.36-4.10) | <0.001 |
| low | Ref (1.00) |  |
| Medium | 2.93 (2.67-3.22) | <0.001 |
| High | 13.84 (12.43-15.42) | <0.001 |
| **Time period** |  |  |
| 2005-2009 | Ref (1.00) |  |
| 2010-2014 | 2.48 (2.15-2.87) | <0.001 |
| 2015-2019 | 2.71 (2.38-3.10) | <0.001 |
| 2020-2024 | 3.31 (2.91-3.77) | <0.001 |

Univariable logistic regression analyses were conducted to evaluate potential risk factors associated with moderate-to-severe acute pancreatitis. Results are presented as unadjusted odds ratios (ORs) with corresponding 95% confidence intervals (CIs) and P-values derived from Wald tests. Reference categories are denoted as "Ref (1.00)".
